# Pressure- vs. volume-controlled ventilation and their respective impact on dynamic parameters of fluid responsiveness: a cross-over animal study

**DOI:** 10.1186/s12871-023-02273-z

**Published:** 2023-09-19

**Authors:** Amelie Zitzmann, Tim Bandorf, Jonas Merz, Fabian Müller-Graf, Maria Prütz, Paul Frenkel, Susanne Reuter, Brigitte Vollmar, Nora A. Fuentes, Stephan H. Böhm, Daniel A. Reuter

**Affiliations:** 1 Department of Anaesthesiology, Intensive Care Medicine and Pain Therapy, University Medical Centre of Rostock, Schillingallee 35, 18057 Rostock, Germany; 2 Rudolf-Zenker Institute for Experimental Surgery, University Medical Centre of Rostock, Rostock, Germany; 3 Department of Research, Hospital Privado de Comunidad, Mar del Plata, Argentina

**Keywords:** Fluid therapy, Hemodynamic monitoring, Fluid responsiveness, Positive pressure respiration, Stroke volume

## Abstract

**Background and goal of study:**

Pulse pressure variation (PPV) and stroke volume variation (SVV), which are based on the forces caused by controlled mechanical ventilation, are commonly used to predict fluid responsiveness. When PPV and SVV were introduced into clinical practice, volume-controlled ventilation (VCV) with tidal volumes (VT) ≥ 10 ml kg^− 1^ was most commonly used. Nowadays, lower VT and the use of pressure-controlled ventilation (PCV) has widely become the preferred type of ventilation. Due to their specific flow characteristics, VCV and PCV result in different airway pressures at comparable tidal volumes. We hypothesised that higher inspiratory pressures would result in higher PPVs and aimed to determine the impact of VCV and PCV on PPV and SVV.

**Methods:**

In this self-controlled animal study, sixteen anaesthetised, paralysed, and mechanically ventilated (goal: VT 8 ml kg^− 1^) pigs were instrumented with catheters for continuous arterial blood pressure measurement and transpulmonary thermodilution. At four different intravascular fluid states (IVFS; baseline, hypovolaemia, resuscitation I and II), ventilatory and hemodynamic data including PPV and SVV were assessed during VCV and PCV. Statistical analysis was performed using U-test and RM ANOVA on ranks as well as descriptive LDA and GEE analysis.

**Results:**

Complete data sets were available of eight pigs. VT and respiratory rates were similar in both forms. Heart rate, central venous, systolic, diastolic, and mean arterial pressures were not different between VCV and PCV at any IVFS. Peak inspiratory pressure was significantly higher in VCV, while plateau, airway and transpulmonary driving pressures were significantly higher in PCV. However, these higher pressures did not result in different PPVs nor SVVs at any IVFS.

**Conclusion:**

VCV and PCV at similar tidal volumes and respiratory rates produced PPVs and SVVs without clinically meaningful differences in this experimental setting. Further research is needed to transfer these results to humans.

## Background

Stroke volume variation (SVV) and pulse pressure variation (PPV) are dynamic parameters commonly used to predict fluid responsiveness. While in former times assessment of the intravascular fluid status was performed using clinical signs, filling pressures and static indices of intravascular volume, more dynamic parameters using heart-lung interactions during controlled mechanical ventilation (CMV) were established with the turn of the millennium [1–3]. CMV with sufficiently high tidal volumes (VT) exerts forces on the intrathoracic vasculature and on the heart resulting in cyclic changes in pre- and afterload for both ventricles with consecutive changes in stroke volume and thus arterial blood pressure [4, 5]. With pulse pressure variation (PPV), derived from arterial waveform analysis via the equation (*maximum pulse pressure – minimum pulse pressure)/mean pulse pressure* within one respiratory cycle [1, 4, 6, 7], and stroke volume variation (SVV), derived from pulse contour analysis via the equation (*maximum stroke volume – minimum stroke volume)/mean stroke volume* within one respiratory cycle [8–11], two parameters were introduced claiming to be predictive of fluid responsiveness [1, 4]. With a given VT and in the absence of arrhythmia, the magnitude of these variations depends on the fluid state only: high values of PPV and SVV indicate positive fluid responsiveness, i.e. a fluid bolus given will result in an increased stroke volume (SV) or cardiac output (CO). The higher the values, the greater the increase in cardiac index (CI) after a fluid bolus administered [6]. Low values are associated with negative fluid responsiveness, but do not predict fluid overload [4, 12]. These findings led to the definition of thresholds to discriminate between responders and non-responders – with a so-called *grey zone*, usually between 9 and 13%, where the behaviour of CI is less predictable or the increase in CI possibly less pronounced than the classical definition of fluid-responsiveness with an increase in SV or CO greater than 15% [1, 10, 13, 14]. Besides the necessity of CMV with VT ≥ 8 ml kg^− 1^ ideal body weight (BW) and a regular heart rhythm, further limitations include a low heart beat-to-respiratory rate-ratio, increased abdominal pressure or open chest-conditions [13, 15]. Furthermore, valvular disease can challenge pulse contour analysis algorithms and thus, the measurement of SV and SVV, especially in extreme grade pathologies, e.g. free aortic regurgitation; within the normal clinical ranges, current algorithms seem to work reliably [16]. Despite all these limitations and the emergence of newer dynamic parameters to guide hemodynamic therapy such as arterial elastance [17, 18], acting independently from tidal volumes or parameters based on tidal elimination of carbon dioxide [19], which have shown promising results, SVV and PPV are widely used as they are available in many hemodynamic monitoring devices [3, 15].

To date, these parameters have been explored in several studies over a range of tidal volumes [5–11, 20], often using volume-controlled ventilation (VCV) which was the ventilation type commonly used at that time [21]. With increasing knowledge about ventilator induced lung injury, pressure-controlled ventilation became the preferred way of ventilation in parts of the world [15, 21]. Due to their specific air flow characteristics VCV and PCV result in different patterns of airway pressures (Paw) and are thus likely to have a different impact on the heart, even if comparable tidal volumes are delivered. In VCV, the primary control variables are the size of the VT and the inspiratory flow rate. A constant flow is delivered, leading to a steady, rather linear increase in airway pressure until peak airway pressure is reached. When the VT has been delivered and inspiratory time is not yet reached, Paw drops to plateau pressure (Pplat) while no flow occurs to or from the ventilator with inspiratory and expiratory valves shut, equilibrating with alveolar pressure [21]. In PCV, inspiratory pressure as the main control variable is maintained throughout the entire inspiratory phase resulting in a square pressure waveform and a decelerating flow pattern. When inspiration time is long enough to reach zero flow, pre-set pressure is in equilibrium with the alveolar pressure at the end of inspiration and equals plateau pressure [21]. Thus, VCV was reported to result in higher peak inspiratory pressures (PIP), but lower mean airway pressures compared to PCV [22, 23].

Since the major cause of SVV and PPV are the changes in positive intra-thoracic pressures generated by positive pressure mechanical ventilation, differences in the pattern of VT application may also result in differences of the cardiovascular effects, specifically in SVV and PPV [24, 25]. Up until now, the magnitude of the tidal volumes has been primarily considered when evaluating the impact of ventilation on SVV and PPV with regards to their ability to assess fluid responsiveness. The control variable, i.e. pressure or volume, however, which was used to generate the respective tidal volumes has been neglected. Therefore, we hypothesised that despite comparable tidal volumes, VCV and PCV would result in different SVV and PPV values at the same fluid state. Thus, the aim of this study was to determine the impact of the two ventilation regimes on the dynamic parameters of fluid responsiveness.

## Methods

The study was approved by the governmental ethics board for animal research (*Landesamt für Landwirtschaft, Lebensmittelsicherheit und Fischerei*, Rostock, Mecklenburg-Vorpommern, Germany; No. 7221.3-1-059/19; veterinarian in charge: Dr Sylvia Hille) on 29/01/2020 and was carried out in accordance with the EU Directive 2010/63/EU and the ARRIVE 2.0 guidelines [26]. Advice from the local institute for biostatistics and informatics was sought for sample size determination. All animals served as their own control; therefore, randomization was not required. Blinding of the investigators was not feasible. After completing all experimental steps, the animals were euthanized in compliance with European and federal laws using pentobarbital (45 mg kg^-1^ intravenously) while maintaining general anaesthesia.

### Anaesthesia and instrumentation

Sixteen German Landrace pigs (12–16 weeks old) were prepared and anaesthetised according to local standards. The animals were brought to the stables of the Institute for Experimental Surgery four to five days before the experiment for acclimatisation. They had free access to food and water until the night prior to the trial. Each animal’s health was checked by the staff of the Central Laboratory Animal Facility on the morning of the trial and admitted only if it showed no abnormalities as defined by local standards. After induction with 200 µg fentanyl, 100 mg propofol and 4 mg pancuronium, a combination of fentanyl (10 µg kg^-1^ h^-1^), propofol (4–8 mg kg^-1^ h^-1^), midazolam (0.1 mg kg^-1^ h^-1^) and pancuronium (6 mg h^-1^) was used for maintenance of anaesthesia to ensure a deep level of sedation and to suppress any spontaneous breathing. After orotracheal intubation with a tube of 7 mm inner diameter, the pigs were mechanically ventilated using the Servo-u ventilator (Getinge AB, Gothenburg, Sweden) to ensure gas exchange during further instrumentation. For assessment of transpulmonary pressures, a NutriVent® nasogastric tube (SIDAM group, Mirandola, Italy) was placed and connected to the auxiliary pressure port of the ventilator. All pigs received a 4 Charrière (Ch) 16 cm PiCCO® (Getinge AB, Gothenburg, Sweden) catheter via the right femoral artery, a 5 Ch high-fidelity pressure sensor catheter (Mikro-Tip® SPR-350, Millar Instruments Inc., Houston, TX, USA) in the descending aorta via the left femoral artery and a central-venous catheter in the right internal jugular vein for hemodynamic monitoring. For induction of hypovolaemia (see below), an 8.5 Ch introducer sheath in the right common carotid artery was used; another 8.5 Ch introducer sheath in the right internal jugular vein was used for re-transfusion and volume challenges. Fluoroscopy was used to verify correct catheter placement.

### Ventilation

Prior the start of the protocol, an automatic stepwise recruitment manoeuvre (Auto SRM) was performed using the ventilator’s built-in function. To prepare the lungs and the cardiovascular system for the following recruitment, PEEP was increased stepwise while keeping the driving pressure (ΔP) constant at 15 cmH_2_O, until an inspiratory pressure of 40 cmH_2_O was reached. This pressure was held over 50 s before proceeding to a decremental PEEP trial with a stepwise (2 cmH_2_O at a time) decrease of PEEP while assessing dynamic respiratory system compliance. With this procedure, the optimal PEEP for each individual pig was defined as the one 2 cmH_2_O above the closing PEEP determined by the ventilator’s built-in “open lung tool”. This individualized PEEP was then used for the duration of the protocol. With this approach, we aimed at inflating the lungs optimally with as few atelectasis as possible to distribute the applied tidal volume evenly within the lungs while achieving the most uniform perfusion without shunt. This way, a maximum impact of ventilation on the heart and vasculature was achieved.

At each fluid state (see below), the same ventilation sequence was performed: after an initial recruitment manoeuvre to ensure equal conditions, the pigs were first ventilated using VCV: VT was set to 8 ml kg^-1^ BW and RR was set to result in an end-tidal partial pressure of carbon dioxide (etCO_2_) between 4.3 and 5.7 kPa, with an inspiration:expiration ratio (I:E) of 1:2 and an inspiratory pause of 30%. When a steady state – defined as changes in tidal elimination of carbon dioxide (VtCO_2_) of less than 10% for one minute – was achieved, hemodynamic and ventilatory data were recorded over a three-minute period. In a second step, ventilation was changed to PCV with inspiratory pressure set to provide again a tidal volume of 8 ml kg^-1^ BW; respiratory rate was again adjusted based on etCO2, and the I:E ratio of 1:2 (without inspiratory pause) remained unchanged. Again, when steady state was achieved, a three-minute period of data was recorded. Before changing the control variable, inspiratory and expiratory hold manoeuvres of ten seconds each were performed to measure total PEEP, Pplat and to derive ΔP.

### Fluid status

For comparison of SVV and PPV at different hemodynamic conditions, four intravascular fluid states (IVFS) were induced:

- Baseline (BL): After instrumentation, a first set of thermodilutions (TD) was performed to assess the intravascular fluid state (IVFS). If SVV was greater than 10%, repeated boluses of colloids (modified gelatine 4% in Ringer’s acetate solution) of 100 ml each were given until SVV remained < 10%. Another set of TD was performed for confirmation. This fluid state was defined as baseline, intended to represent normovolaemia.
- Hypovolaemia (Hypo): In this step, 25 ml kg^-1^ of whole blood were to be withdrawn while the blood was collected in blood bags with heparin to avoid clotting. In case of severe hemodynamic instability, blood withdrawal was paused to allow for hemodynamic stabilization, was stopped if considered too dangerous or resumed when deemed appropriate by the principal investigator. Hemodynamic instability was defined as the combination of non-displayable blood pressure or continuous cardiac output via the femoral arterial line by the PulsioFlex® Monitor in combination with a decrease in end-tidal CO_2_ by more than 50%.
- Resuscitation stage I (Res I): A total of 50% of the previously withdrawn blood was re-transfused.
- Resuscitation stage II (Res II): In addition to the remaining half of the withdrawn blood, 20 ml kg^-1^ of colloids were infused.

At each fluid state, after the respective recording of data during VCV and PCV, a fluid challenge (FC) was performed to assess volume responsiveness, defined as an increase in cardiac index (CI) by at least 15%: during Hypo and Res I, 7 ml kg^-1^ of the withdrawn blood were used as fluid bolus, during BL and Res II, the same amounts of colloids were infused.

### Outcome variables

Primary outcomes were differences in PPV and SVV during VCV and PCV. As secondary outcomes, airway and transpulmonary pressures as well as arterial and central venous pressures between the two ventilation patterns were assessed.

### Data recording and processing

Hemodynamic data were recorded at a sampling rate of 10 kHz using bridge transducer amplifiers in combination with the respective hard- and software PowerLab 16/35 and LabChart 8 (both ADInstruments, Dunedin, New Zealand). Cardiac output was measured via transpulmonary thermodilution (TPTD) and recorded using the PulsioFlex® system (Getinge AB, Gothenburg, Sweden), ventilator data were recorded using the ServoTracker® software (Getinge AB, Gothenburg, Sweden). At each protocol step recorded data were checked for phases of arrhythmias, which were excluded before representative one-minute periods of good quality data were chosen for further analysis. Data analysis was performed with LabChart and Matlab (MathWorks®, Natick, MA, USA).

### Statistics

Statistical analysis was performed using SigmaPlot 13.0 (Systat Software, Palo Alto, CA, USA) and Stata/IC 15.1 for MAC. Hemodynamic and ventilation parameters are displayed as median [interquartile range (IQR)]. Mann-Whitney Rank Sum Test was performed to test for differences between the two ventilation forms. Friedman Repeated Measures One Way Analysis of Variance on Ranks (RM ANOVA on ranks) was used to test for differences between the fluid states; to isolate the group(s) that differ from the others, a Tukey test was used for all pairwise multiple comparison procedures. To evaluate the effect of the ventilatory patterns (VCV and PCV) on SVV and PPV during the four IVFS, a descriptive longitudinal data analysis (LDA) of the determinations made at each moment of the study protocol was performed. Total, between-subject and within-subject variability for the variables of interest are described. To estimate the effect of ventilatory pattern (VCV or PCV) on PVV and SVV, we used *Generalized Estimating Equations* (GEE) including the four IVFS as the independent variable *(T: timepoints)*, with a normally distributed probability function, an identity link, and an intersection of random effects to account for the correlation between observations within the repeated measures model. To select the working correlation structure, a “naive” linear regression analysis was first carried out, assuming that within-subjects’ observations are independent. Then, based on the residuals from this analysis, the parameters of the working structure of the correlation were calculated. Based on this, an exchangeable structure of work correlation was selected. Beta Coefficients with their 95% Confidence Intervals (CI95) are presented for LDA. *P* ≤ 0.05 was considered statistically significant.

## Results

Sixteen animals were included in the study. Three animals died during instrumentation due to arrhythmias caused by intraventricular catheter placement. Five animals died during induction of hypovolaemia or during the following measurements. Finally, complete data sets of eight pigs were recorded, analysed, and are reported here.

The animals weighed 40.3 [30.8–43.0] kg. For induction of severe hypovolaemia, 826 [425–1069] ml corresponding to 22.5 [14–25] ml kg^-1^ of blood were withdrawn.

### Ventilatory data

The automatic stepwise recruitment manoeuvre revealed a PEEP of 10 cmH_2_O as optimal for all but one animal, which had a PEEP of 8 cmH_2_O.

**Fig. 1:**
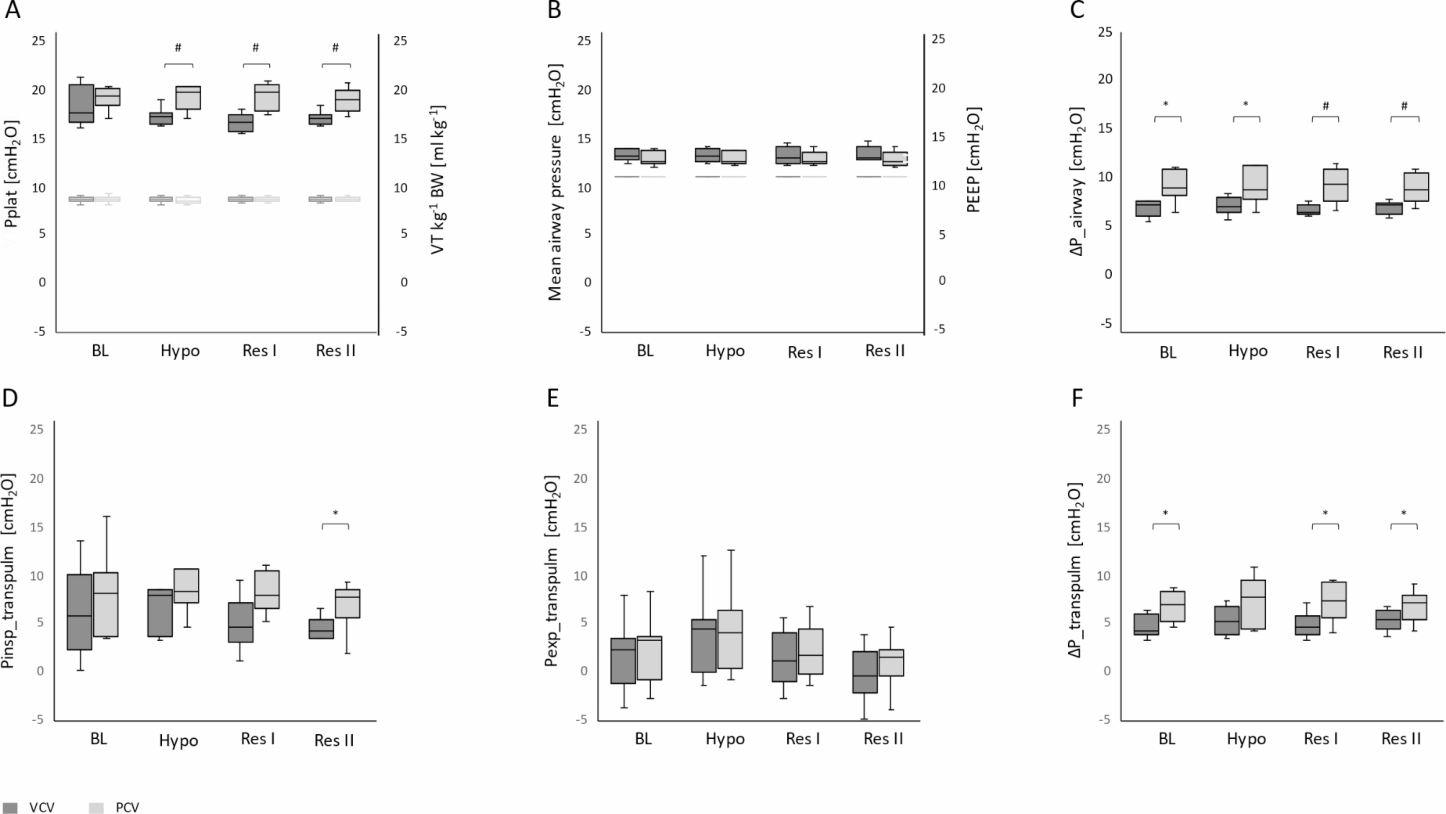
Airway **(A – C)** and transpulmonary **(D – F)** pressures during Volume- and Pressure-Controlled Ventilation. Boxplots with median, IQR and range; #: P < 0.01; *: P < 0.05. In A, the respective tidal volumes can be seen in the lower part of the diagram together with the corresponding plateau pressures. In B, the upper row depicts mean airway pressure and the lower row PEEP. Pplat, plateau pressure; VT kg^− 1^BW, tidal volume per kilogram bodyweight; PEEP, positive endexpiratory pressure; ΔP_airway, airway driving pressure; Pinsp_transpulm, inspiratory transpulmonary pressure; Pexp_transpulm, expiratory transpulmonary pressure; ΔP_transpulm, transpulmonary driving pressure; BL, baseline; Hypo, hypovolaemia; Res I, resuscitation stage I; Res II, resuscitation stage II.

Measured VTs were within 95% of the desired range and with 7.8 [7.6–7.9] ml kg^-1^ for VCV and 7.7 [7.5–8.0] ml kg^-1^ for PCV comparable (*P = 0.69)*. At identical mean airway pressures and respiratory rates, VCV generated significantly higher peak inspiratory pressures than PCV in all IVFS (*P ≤ 0.01*). A comparison of airway and transpulmonary pressures at the different IVFS is presented in Fig. 1 and in Table 1.

**Table 1:** Airway and transpulmonary pressures at 8 ml kg^-1^ between the two ventilation regimes

Ventilation parameter	Fluid state	VCV	PCV	*P*
Peak Inspiratory Pressure [cmH_2_O]	Baseline	22.3 [20.6–25.1]	18.5 [17.5–19.2]	***0.005***
	Hypovolaemia	23.4 [20.7–25.3]	18.7 [17.1–19.3]	***0.005***
	Resuscitation I	23.1 [20.4–25.0]	18.9 [16.9–19.5]	***0.010***
	Resuscitation II	23.6 [21.2–25.4]	18.0 [16.8–19.0]	***< 0.001***
Plateau Pressure [cmH_2_O]	Baseline	16.7 [15.7–19.5]	18.5 [17.5–19.2]	*0.195*
	Hypovolaemia	16.3 [15.4–16.6]	18.7 [17.1–19.3]	***0.005***
	Resuscitation I	15.6 [14.8–16.4]	18.9 [16.9–19.5]	***0.010***
	Resuscitation II	16.1 [15.4–16.5]	18.0 [16.8–19.0]	***0.003***
Driving Pressure [cmH_2_O]	Baseline	7.2 [5.9–7.5]	9.0 [8.2–10.8]	***0.050***
	Hypovolaemia	6.9 [6.4–7.9]	8.7 [7.8–11.2]	***0.015***
	Resuscitation I	6.5 [6.1–7.1]	9.4 [7.6–10.9]	***0.002***
	Resuscitation II	7.2 [6.2–7.5]	8.8 [7.6–10.4]	***0.005***
Transpulmonary Inspiratory Pressure [cmH_2_O]	Baseline	4.8 [1.3–9.2]	7.1 [2.7–9.3]	*0.383*
	Hypovolaemia	7.0 [2.7–7.5]	7.3 [6.2–9.7]	*0.383*
	Resuscitation I	3.6 [2.4–5.4]	6.8 [4.2–9.3]	*0.053*
	Resuscitation II	3.3 [2.6–4.5]	6.8 [4.7–7.5]	***0.026***
Transpulmonary Expiratory Pressure [cmH_2_O]	Baseline	1.4 [-2.0–2.5]	2.3 [-1.6–2.8]	*0.456*
	Hypovolaemia	3.6 [-0.9–4.5]	3.2 [-0.6–5.5]	*0.902*
	Resuscitation I	0.3 [-1.8–3.2]	0.8 [-1.1–3.6]	*0.589*
	Resuscitation II	-1.4 [-3.0–1.2]	0.6 [-1.4–1.3]	*0.318*
Transpulmonary Driving Pressure [cmH_2_O]	Baseline	3.3 [2.9–5.1]	6.1 [4.3–7.4]	***0.038***
	Hypovolaemia	4.2 [2.9–5.9]	6.9 [3.5–8.5]	*0.065*
	Resuscitation I	3.8 [2.9–4.8]	6.4 [4.7–8.3]	***0.021***
	Resuscitation II	4.5 [3.5–5.5]	6.2 [4.5–6.9]	***0.050***

*Data are presented as median [IQR]. VCV, volume-controlled ventilation; PCV, pressure-controlled ventilation. P < 0.05 is considered statistically significant and respective values are printed in bold.*

### Hemodynamics

Blood withdrawal led to a decrease of the global enddiastolic volume index (GEDI) from 462 [416–516] ml m^-2^ at baseline to 324 [299–343] ml m^-2^ (hypovolaemia); the first resuscitation step increased it to 366 [313–407] ml m^-2^ and to 477 [437–502] ml m^-2^ after the second resuscitation manoeuvre. TD-derived Baseline-Cardiac Index was 3.32 [2.90–3.74] l min^-1^ m^-2^ and decreased to 1.76 [1.39–2.03] l min^-1^ m^-2^ after bleeding. With the resuscitation manoeuvres it increased to 2.40 [1.93–2.80] l min^-1^ m^-2^ and 3.84 [3.37–4.08] l min^-1^ m^-2^, respectively.

**Fig. 2:**
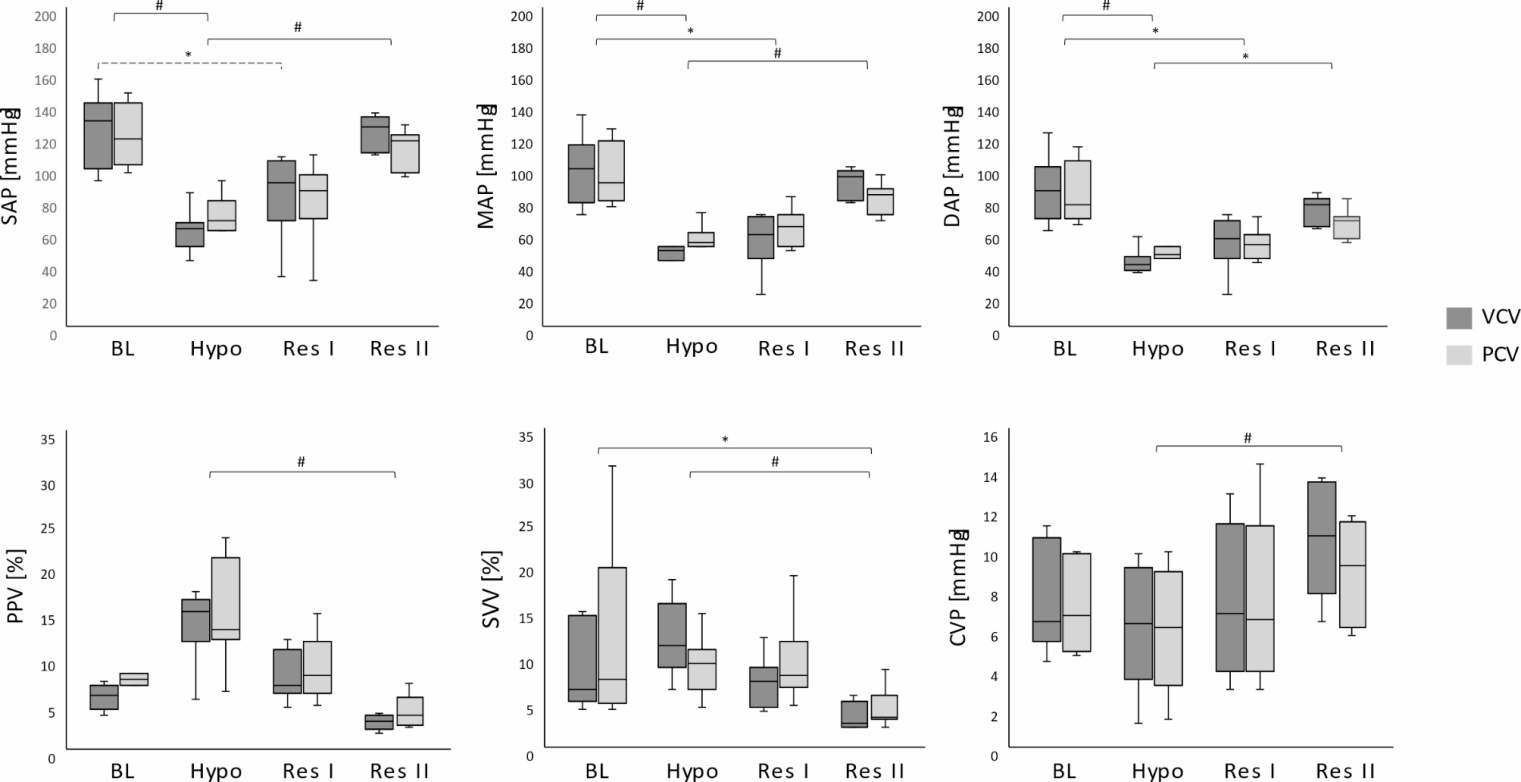
Arterial and central venous pressures, Pulse Pressure Variation and Stroke Volume Variation. Boxplots with median, IQR and range; #: P < 0.01; *: P < 0.05; broken-lined brackets represent differences between the individual fluid states for VCV only; solid-lined brackets represent differences for both ventilation regimes.SAP, systolic arterial pressure; MAP, mean arterial pressure; DAP, diastolic arterial pressure; PPV, pulse pressure variation; SVV, stroke volume variation; CVP, central venous pressure; BL, baseline; Hypo, hypovolaemia; Res I, resuscitation stage I; Res II, resuscitation stage II.

The courses of arterial and central venous pressures during the interventions together with the respective PPV and SVV values are shown in Table 2 and in Fig. 2. Arterial pressures decreased significantly (*P < 0.01*) during bleeding and rose stepwise after re-transfusion/infusion. PPV and SVV increased markedly but not significantly after volume depletion and dropped again to reach values below baseline after the second resuscitation step, which were significantly different from those at hypovolaemia (*P < 0.01*).

**Table 2:** Hemodynamic parameters at the respective fluid states compared between volume- and pressure-controlled ventilation

Hemodynamic parameter	Fluid state	VCV	PCV	*P*
**Heart rate [bpm]**	Baseline	109 [89–115]	105 [92–114]	*0.798*
	Hypovolaemia	121 [106–164]	121 [99–157]	*0.645*
	Resuscitation I	109 [98–120]	95 [91–116]	*0.234*
	Resuscitation II	93 [88–113]	92 [88–108]	*0.645*
**Systolic pressure [mmHg]**	Baseline	129 [98–139]	117 [101–140]	*0.878*
	Hypovolaemia	61 [49–64]	66 [60–78]	*0.382*
	Resuscitation I	89 [66–104]	84 [67–94]	*0.798*
	Resuscitation II	124 [108–131]	115 [96–120]	*0.195*
**Mean arterial pressure [mmHg]**	Baseline	99 [77–113]	89 [79–115]	*0.959*
	Hypovolaemia	47 [41–50]	53 [50–59]	*0.083*
	Resuscitation I	58 [42–68]	62 [50–69]	*0.505*
	Resuscitation II	93 [78–97]	81 [69–85]	*0.279*
**Diastolic pressure [mmHg]**	Baseline	85 [66–100]	76 [67–103]	*0.878*
	Hypovolaemia	39 [35–43]	45 [42–49]	*0.130*
	Resuscitation I	54 [42–66]	51 [42–57]	*0.645*
	Resuscitation II	78 [62–80]	66 [54–69]	*0.279*
**Central venous pressure [mmHg]**	Baseline	6.3 [5.3–10.5]	6.6 [4.8–9.7]	*0.505*
	Hypovolaemia	6.2 [3.3–9.0]	6.0 [3.1–8.8]	*0.721*
	Resuscitation I	6.7 [3.8–11.2]	6.4 [3.7–11.1]	*0.878*
	Resuscitation II	10.6 [7.7–13.3]	9.1 [5.9–11.3]	*0.161*
**Cardiac index** ^+^ **[l min** ^**-1**^ **m** ^**-2**^ **]**	Baseline	3.32 [2.90–3.74]	3.08 [2.72–3.62]	*0.645*
	Hypovolaemia	1.76 [1.39–2.03]	1.66 [1.17–2.18]	*0.798*
	Resuscitation I	2.40 [1.93–2.80]	2.46 [1.90–2.80]	*0.878*
	Resuscitation II	3.84 [3.37–4.08]	3.70 [3.47–3.98]	*0.721*
**Pulse pressure variation** **[%]**	Baseline	5.9 [4.2–7.0]	7.7 [7.0–8.2]	< 0.001*
	Hypovolaemia	15.1 [11.8–16.4]	13.1 [12.0–20.9]	
	Resuscitation I	7.0 [6.1–10.8]	8.1 [6.0–11.7]	
	Resuscitation II	2.9 [2.2–3.7]	3.6 [2.4–5.7]	
**Stroke volume variation [%]**	Baseline	6.2 [4.9–14.4]	7.3 [4.8–19.6]	< 0.001*
	Hypovolaemia	11.2 [8.6–15.8]	9.0 [6.4–10.7]	
	Resuscitation I	7.2 [4.3–8.6]	7.7 [6.6–11.5]	
	Resuscitation II	2.5 [2.0–5.0]	3.2 [3.0–5.5]	

*Data are presented as median [IQR]. VCV, volume-controlled ventilation; PCV, pressure-controlled ventilation; bpm, beats per minute. P < 0.05 is considered statistically significant.*^*+*^pulse-contour derived cardiac index. * association between changes in PPV and in SVV during VCV and PCV was determined by longitudinal data analysis (LDA).

None of the abovementioned hemodynamic parameters showed any differences between VCV and PCV at any IVFS, nor did pulse-contour-derived cardiac outputs.

LDA of PPV and SVV over the respective IVFS revealed strongly balanced and complete data. The variability in the data was lower between subjects than the overall variability within subjects (Table 3) for PPV and SVV. The change over time of the hemodynamics parameters was similar in both ventilation types. An association between changes in PPV (p < 0.001) in VCV and PCV [coefficient of PPV 1.111 (CI95 0.821–1.400)], and likewise between SVV (p < 0.001) in VCV and PCV [coefficient 0.6317 (CI95 0.430–0.832)] can be seen.

**Table 3:** Overall, between-subject and within-subject variability of PPV and SVV

Variable	VCV			PCV			
	Mean	Standard deviation		Mean	Standard deviation		
PPV	7.771	overall	4.726	9.201	overall	6.527	
		between	1.917		between	2.414	
		within	4.360		within	6.110	
SVV	8.312	overall	6.393	8.475	overall	6.076	
		between	3.778		between	2.549	
		within	5.289		within	5.572	
with N = 32, n = 8 and T = 4							

N, number of measurements, n, number of pigs, T, number of timepoints

## Discussion

We aimed to investigate if the different types of gas inflation during VCV and PCV would result in different PPV- and SVV-values despite comparable VT. In our experimental setting, arterial and central venous pressures showed the expected physiological behaviour during the respective IVFS [27]. PPV and SVV showed the predictable and physiological increase, indicating incremental fluid responsiveness after blood was removed, as described in previous studies [1, 6, 8, 10]. Hypovolaemia was confirmed by the decrease in GEDI [27, 28]. During the hypovolaemic state, PPV values were above the clinically accepted thresholds [13, 14], thus indicating fluid responsiveness, which was confirmed by an increase in CI after the fluid challenge. However, after the first resuscitation, PPV dropped to values below the thresholds, thus contradicting fluid responsiveness despite an increase in CI after the fluid challenge [6]. After the second resuscitation, PPV values dropped even below baseline values. This is a finding also observed in a study by Fujita and co-workers [29], but does not mandatorily happen during over-infusion, as observed by Taguchi et al. [12]. Thus, no inferences on the volaemic state should be drawn from low values, but on fluid responsiveness. For SVV, values at the hypovolaemic state changed less than PPV values, which has been described before and is thought to be due to physiological and measurement reasons [27, 30]. Values at the hypovolaemic state were within the grey zone albeit the marked increase in CI after the fluid challenge. PCV produced significantly higher PPV values than VCV, whereas SVV values were significantly lower during PCV. Nevertheless, with correlation coefficients of 1.11 and 0.63, the observed differences should not be of clinical significance as they represent less than 1% in absolute terms of the typical PPV- and SVV-values. There were significant differences between the airway pressures. In accordance with previous studies [22, 23], we found significant differences in peak inspiratory pressures, which were systematically higher during VCV due to the flow-resistive pressure drop caused by the constant flow pattern. In contrast, plateau pressures and thus, the resulting driving pressures were lower during VCV compared to PCV [22, 23]. However, these pressures are measured within the ventilator, but are presumably not the ones effective at the intrathoracic level. It has been reported that only 70% of pressures applied by the ventilator are transmitted to the juxtacardiac pleura; the percentage of transmission to the pericardium and the vena cava is even lower (37% and 43%, respectively) [31]. Also, in our setting, transpulmonary driving pressures, assessed with the help of an oesophageal catheter, were 50–80% of the applied airway driving pressures. Furthermore, the characteristic airway pressure curves with the peak at early inspiration dropping to a plateau at VCV were blunted in the transpulmonary pressure curves [31], resulting in similar pressure patterns as the PCV curves, with non-significantly differences in the transpulmonary inspiration pressures in our experimental setting. Landsdorp and co-workers reasoned that tidal volumes are more relevant for the generation of heart-lung-interactions than the respective (airway) pressures as compliance of the respiratory system influences the transmission of airway pressures to the intrathoracic cavities such as the pleura and the pericardium [31]. In our experimental setting, identical VT generated by different patterns of gas delivery induced differences in airway pressures, which resulted in small but significant differences in PPV and SVV. Lower airway and transpulmonary driving pressures during VCV correlated with higher SVV-values compared to PCV despite identical VT. However, this was not the case for PPV-values, which during VCV tended to be lower at lower driving pressures. Whether this was caused by differences in the interaction with the heart and the vasculature of the pattern of gas delivery or different proportions of ventilation pressures transmitted to the cardio-vascular system [31] cannot be answered from the available data.

### Limitations

This is an experimental study and results cannot be transferred to humans without limitations. We investigated adolescent animals without any known cardiovascular or pulmonary pathology. Lung compliance was therefore most probably higher than in adult humans. Furthermore, this protocol did not use vasopressors to compensate for hypovolaemic hypotension as clinicians would normally do to bridge the time until enough fluids have been administered. As we wanted to investigate physiological responses of SVV and PPV to different ventilation regimes and fluid states without exogenously induced changes in arterial compliance, vasopressors were omitted. As every pig served as its own control with VCV and PCV measurements being performed at one fluid state, fluid responsiveness using a volume challenge could not be assessed separately for each ventilation regime. To compensate for this, VCV measurements, which have been validated in previous studies, were performed first, followed by PCV measurements. After that, ventilation was switched back to VCV and another set of data was analysed to detect changes during the measurements, before performing the volume challenge. At all fluid states, there were no significant differences neither in hemodynamic nor in ventilation parameters between the first and the second VCV sets. After the first eight pigs – of which four had not survived the volume depletion – the protocol was adjusted, and blood withdrawal was limited to increase chances of survival. Sample size calculation was not feasible for this study; therefore, data analysis was performed irrespective of the total number, also to meet animal research standards.

## Conclusion

Aiming at identical tidal volumes, pressure-controlled and volume-controlled ventilation resulted in different airway pressures. These resulted in opposing behaviours of PPV and SVV. However, these differences might not be clinically meaningful despite statistical significance. Further research is needed to suggest whether volume- or pressure-controlled ventilation at identical tidal volumes should be used to determine stroke volume variation and pulse pressure variation for guiding fluid therapy in humans.

## Data Availability

The datasets used and/or analysed during the current study are available from the corresponding author on reasonable request.

## Abbreviations

ARRIVE: Animal Research Reporting of In Vivo Experiments
Auto SRM: automatic stepwise recruitment manoeuvre
BL: baseline
BW: body weight
CI: cardiac index
CMV: controlled mechanical ventilation
etCO_2_: endtidal partial pressure of carbon dioxide
FC: fluid challenge
GEDI: global enddiastolic volume index
Hypo: hypovolaemia
I:E: inspiration expiration ratio
IQR: interquartile range
IVFS: intravascular fluid state
P: probability
Paw: airway pressure
PCV: pressure-controlled ventilation
PEEP: positive end-expiratory pressure
PIP: peak inspiratory pressure
PPV: pulse pressure variation
Res I: resuscitation stage I
Res II: resuscitation stage II
RM ANOVA: Repeated Measures One Way Analysis of Variance
RR: respiratory rate
SVV: stroke volume variation
TD: thermodilution
TPTD: transpulmonary thermodilution
VCV: volume-controlled ventilation
VT: tidal volume
ΔP: driving pressure
